# Central emotions and hubs in a colexification network

**DOI:** 10.1038/s41598-023-48922-8

**Published:** 2023-12-09

**Authors:** Mitsuki Fukuya, Tomoko Matsumoto, Yutaka Shimada, Tohru Ikeguchi

**Affiliations:** 1 Department of Information and Computer Technology, Tokyo University of Science, 6-3-1 Niijuku, Katsushika-ku, 125-8585 Tokyo Japan; 2 Institute of Arts and Sciences, Tokyo University of Science, 1-3 Kagurazaka, Shinjuku-ku, 162-8601 Tokyo Japan; 3 Department of Information and Computer Sciences, Saitama University, 255 Shimo-okubo Sakura-ku, Saitama-shi, 338-8570 Saitama Japan

**Keywords:** Computational science, Information technology

## Abstract

By focusing on colexification, we detected central emotions sharing semantic commonalities with many other emotions in terms of a semantic relationship of both similarity and associativity. In analysis, we created colexification networks from multiple languages by assigning a concept to a vertex and colexification to an edge. We identify concepts of emotions with a large weight in the colexification network and specify central emotions by finding hub emotions. Our resultant central emotions are four: “GOOD,” “WANT,” “BAD,” and “LOVE.”

## Introduction

Emotions have a significant influence on human decision-making and behavior. The study of the semantics of emotion words has a rich history in the fields of psychology and linguistics. This research includes both qualitative semantic analyses (e.g., Wierzbicka, 1992^1^, 1999^2^ and Johnson-Laird et al., 1989^3^) and quantitative analysis based on semantic similarity ratings (e.g., Russell, 1980^4^). Recently, unlike these traditional studies, Jackson et al. introduced a novel strategy for the classification of emotions using a colexification dataset from a perspective of network theory^5^.

Colexification is the phenomenon of a single word associated with multiple concepts having assumed semantic relationships^6^. For example, the Spanish word “malo” can have two meanings “BAD” and “SEVERE”; thus, the two concepts, “BAD” and “SEVERE,” are colexified in Spanish. Colexification indicates that the concepts have relevance, including similarity and associativity, which might generate perceived covariation and co-occurrence^7^. Colexification analysis is a newer linguistic method of indirect semantic similarity analysis that has the advantage of not requiring the collection of similarity data from subjects, because it is based on an existing semantic relation (known from translation dictionaries and the like). Jackson et al. focused on the concepts of emotions (emotional concepts) and identified groups of the emotional concepts by a cluster analysis based on the similarity between the emotional concepts in the relevance^5^. However, Jackson et al. only compared languages through the identified groups of the emotional concepts in the colexification network, and they did not sufficiently discuss the relationship between emotions and features of vertices corresponding to the emotional concepts in the colexification network.

Therefore, in this study, we attempted to clarify the relationship between the structure of a colexification network and emotions by identifying hubs in the colexification network. We created a colexification-based network, where vertices represent concepts of emotions, and two vertices are connected if their corresponding concepts are directly or indirectly colexified. A vertex connected with many edges in a network is termed as a hub in the network theory. In a network, emotions corresponding to hub vertices are semantically related to many other emotions. We identified hub vertices within the colexification network, which we refer to as *“central emotions.”* We will later discuss how the central emotions differ from the traditional concept of basic emotions, thereby elucidating the significant emotions within a colexification network. As a result, we extracted four central emotions as follows: “GOOD,” “WANT,” “BAD,” and “LOVE.”

Estimating central emotions through network theory offers a distinct advantage, as it allows us to identify emotions evoked not only by similar emotions but also dissimilar emotions that are linked to each other associatively or by cooccurence (e.g. Kuppens et al., 2004^7^). Hence, central emotions hold significance not only within relationships based on similarity, but also across relationships that span different groups.

## Methods

### Creating a network of colexification

**Figure 1:**
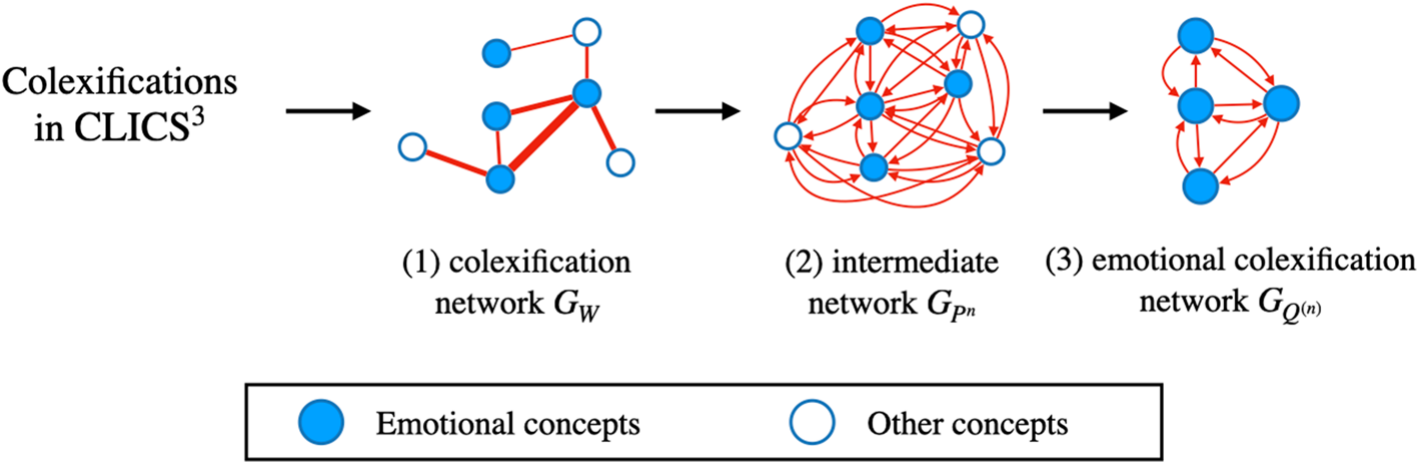
How to generate an emotional colexification network for our analysis.

Figure 1 shows a flow of how to generate an emotional colexification network.

**Figure 2:**
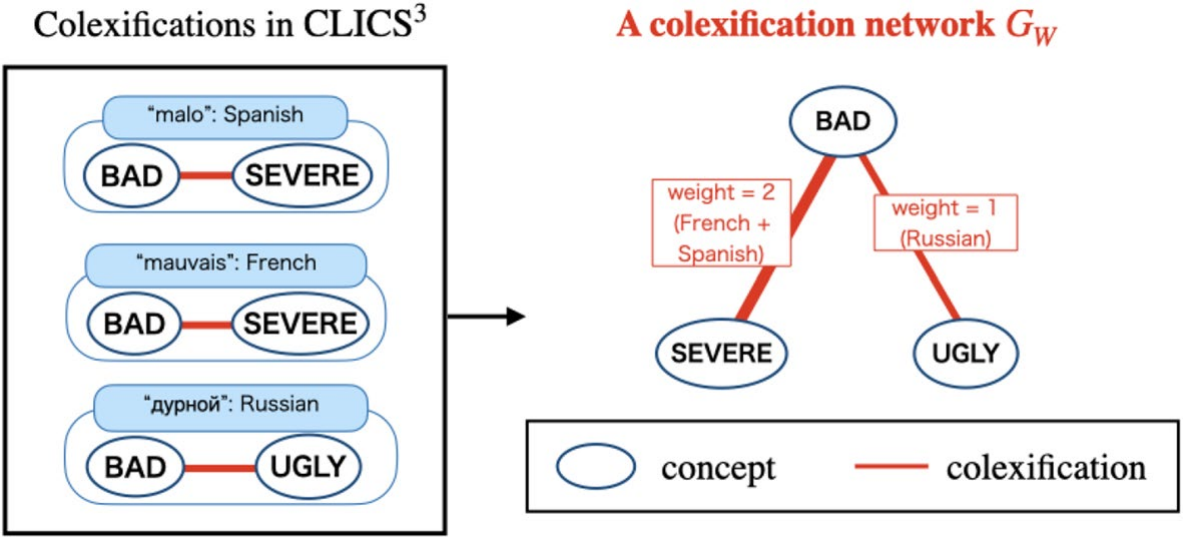
How to define an edge weight in the colexification network $G_W$.

First, we created a weighted adjacency matrix $W=(w_{ij})$ using all concepts in $\textrm{CLICS}^3$^8^ and defined a colexification network $G_{W}$, whose weighted adjacency matrix is *W* (Fig. 1(1)). In the colexification network $G_{W}$, the vertices represent concepts. Two vertices are connected by a weighted edge when their corresponding concepts are colexified in one or more languages. Let $c_{i}$ be a concept corresponding to vertex *i* and $w_{ij}$ be the weight of the edge connecting vertices *i* and *j*, which corresponds to the number of languages in which two concepts $c_{i}$ and $c_{j}$ are colexified. Figure 2 shows how to define an edge weight in a colexification network. An example in Fig. 2 uses three concepts “BAD,” “SEVERE,” and “UGLY,” which are colexified in Spanish, French, and Russian.

In Fig. 2, for example, the weight of the edge connecting “BAD” and “SEVERE” is two because “BAD” and “SEVERE” are colexified in two languages, French and Spanish. Concepts connected by edges with large weights tend to be colexified in many languages and more relevant to each other.

Second, we created an intermediate network $G_{P^{n}}$ (Fig. 1(2)). In Fig. 2, no edges are drawn between “SEVERE” and “UGLY”; however, both these concepts and “BAD” are colexified in three languages. Therefore, “SEVERE” and “UGLY” have an indirect connection through the colexification associated with “BAD.”

To quantify this indirect relationship and clarify relationships between distant vertices, we applied a random walk approach to the colexification network. In our random walk approach, random walkers move along edges based on the transition probability matrix $P=(p_{ij})$, where $p_{ij}$ is the probability of a random walker on vertex *i* moving to vertex *j* in network $G_W$. In a network $G_W$ with $N_W$ vertices, $p_{ij}$ is defined as $p_{ij} = w_{ij}/ \sum _{s=1}^{N_W}w_{is}$. The transition probability matrix after *n* steps is $P^{n}$, and the (*i*, *j*)th element of $P^{n}$, namely $p_{ij}^{n}$, shows the indirect relationship between concepts $c_{i}$ and $c_{j}$. The transition probability matrix is expected to converge as *n* increases mathematically, and then $P^{n}$ satisfies $p_{ij}^{n}=\pi _{j}$ for a sufficiently large *n*, where $\pi _{j}$ is the probability of being at vertex *j* and satisfies $\sum _{j=1}^{N_{W}}\pi _{j}=1$.

In the following, we set $n=10^{3}$, where $p_{ij}^{n}$ nearly converged. We created a network $G_{P^{n}}$ whose directed weighted adjacency matrix is $P^{n}$. The network $G_{P^{n}}$ is a directed network as the edge weights are transition probabilities, and it quantifies the indirect relationships between vertices. In a network $G_{P^{n}}$, $p_{ij}^{n}$ shows the relevance of the vertex *i* to the vertex *j* as the edge weights. Moreover, if $p_{ij}^{n}$ with a large *n* has a large value, a random walker in the network $G_{P^{n}}$ will reach vertex *j* from vertex *i* after passing through many vertices. In other words, the concept $c_{j}$ is likely to be associated with the concept $c_{i}$ via many concepts when $p_{ij}^{n}$ has a large value.

Finally, we created an emotional colexification network $G_{Q^{(n)}}$ (Fig. 1(3)). We defined 25 typical emotional concepts based on the definitions of typical emotions from a previous study^5^ (see also Sec. S1 in SI text for details). Extracting the 25 typical emotional concepts and edges that connect them from the network $G_{P^{n}}$, we generated a $25\times 25$ weighted adjacency matrix $Q^{(n)}=(q_{ij}^{(n)})$ by deleting rows and columns corresponding to concepts that are not the 25 typical emotional concepts. By focusing on emotional concept $c_{j}$ when $q_{ij}^{(n)}$ is large in a network $G_{Q^{(n)}}$, we identify the emotional concepts having a strong relevance to other emotional concepts. We defined hubs in a network $G_{Q^{(n)}}$ from meaning-intensive emotional concepts.

### Specifying hub vertices

In this study, we used the emotional colexification network that is a directed weighted network. Thus, we defined a hub as a vertex whose sum of weights of incoming edges is large. An incoming edge connecting to vertex *i* in the emotional colexification network indicates the strength of relevance in terms of other concepts. We defined the strength of incoming edges of a vertex *i*, denoted by $\textrm{In}_{i}^{(n)}$, as follows: $\textrm{In}_{i}^{(n)}=\sum _{j=1, j\ne i}^{N_{Q^{(n)}}}q_{ji}^{(n)}$, where $N_{Q^{(n)}}$ is the number of vertices in $G_{Q^{(n)}}$, $q_{ji}^{(n)}$ is the probability that a random walker on vertex *j* moves to vertex *i* after *n* steps, and $\pi _{i}$ is the probability of being at vertex *i*. If $q_{ji}^{(n)}$ converges to $\pi _{i}$ in any *j* for $n\rightarrow \infty$, $\textrm{In}_{i}^{(n)}$ is described as follows: $\textrm{In}_{i}^{(n)} = (N_{Q^{(n)}}-1)\pi _{i}$. The strength $\textrm{In}_{i}^{(n)}$ is proportional to the probability of reaching a vertex *i* from all vertices in an emotional colexification network $G_{Q^{(n)}}$. In other words, the strength $\textrm{In}_{i}^{(n)}$ is associativity based on the relevance of a concept corresponding to vertex *i* to all concepts.

We also defined $r^{(n)}_{i}$ as the rank of vertex *i* in the descending order of $\textrm{In}_{i}^{(n)}$, and the rate of change in $\textrm{In}_{i}^{(n)}$ is denoted by $\theta _{i}^{(n)}$, as follows: $\theta _{i}^{(n)}=\textrm{In}_{i}^{(n)}/\textrm{In}_{h}^{(n)}$, where vertex *h* has a rank $r_{h}^{(n)}$ satisfying $r_{h}^{(n)}=r_{i}^{(n)}+1$.

We defined hubs in the emotional colexification network $G_{Q^{(n)}}$ as vertices with large $\textrm{In}_{i}^{(n)}$ and the largest $\theta _{i}^{(n)}$. Concepts corresponding to hubs in network $G_{Q^{(n)}}$ are considered to be abstract and have many elements in common with other emotional concepts. We interpret these concepts as central emotions from the perspective of language using colexification.

### Investigating relationships of emotions

The network $\,G_{Q^{(n)}}\,$ shows not only direct but also indirect relationships as weights of edges. However, the direct relationships have stronger relevance than indirect relationships. To represent both of these direct and indirect relationships as edge weights in the network, a directed weighted adjacency matrix $\,R^{(n)}\,$ is defined as follows: $\,R^{(n)}=\sum _{s=1}^{n}\alpha ^{s}Q^{(s)}\,$. The elements of $\,Q^{(n)}\,$ are always larger than or equal to 0. Therefore, $\alpha =0.8$, so that the values of the elements of *R* converge as *n* increases. We define a network $G_{R^{(n)}}$ whose directed weighted adjacency matrix is $R^{(n)}$, and call $G_{R^{(n)}}$ an aggregated emotional colexification network.

To extract a set of emotional concepts densely connected to each other in the aggregated emotional colexification network $G_{R^{(n)}}$, we used the cluster optimal community detection algorithm (cluster_optimal in R-igraph)^9^. Emotional concepts belonging to the same community are strongly related to each other. Then, we defined the result of community detection of emotional concepts as the discrete classification of emotions based on the similarity of emotions. To show the relationship between the results of community detection and hubs in the emotional colexification network $G_{R^{(n)}}$, the emotional concepts with high relevance to the emotional concepts of the hubs were stratified by hierarchical clustering of edge weights.

## Results

**Figure 3:**
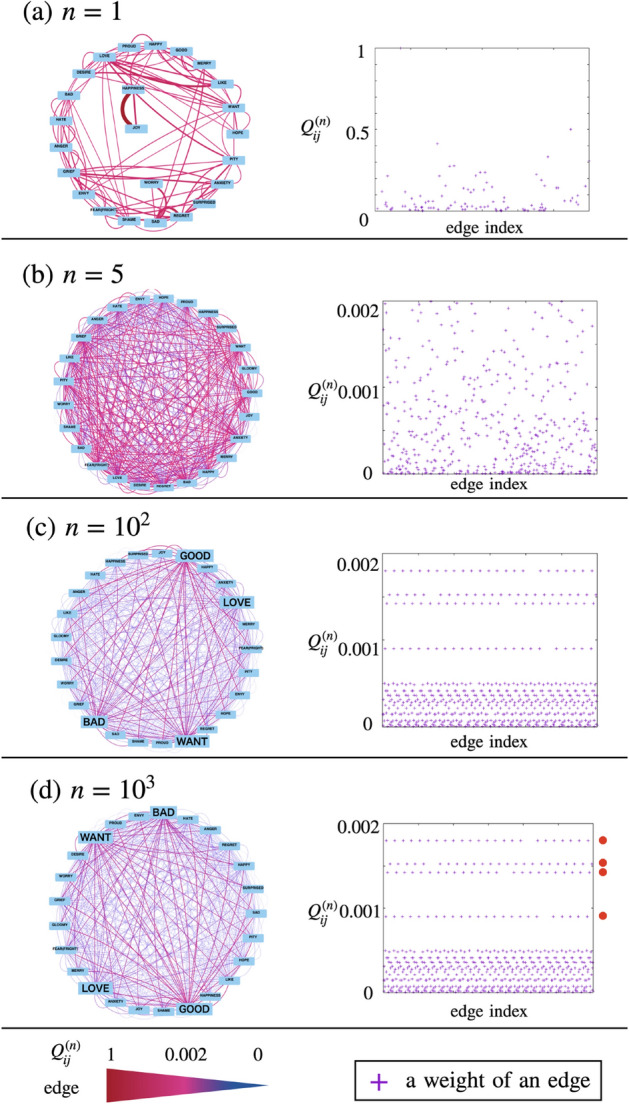
Emotional colexification networks and distributions of edge weights when $n=1,5,10^{2},$ and $10^{3}$. The left panel shows emotional colexification networks $G_{Q^{(n)}}$. The width of an edge in the networks becomes large as the edge weight increases. The color of an edge in the networks changes to red from blue as the edge weight increases. Four vertices corresponding to emotional concepts – “GOOD,” “WANT,” “BAD,” and “LOVE” – are connected by red edges with large weights in the network $G_{Q^{(10^{3})}}$. The right panel plots of distributions of edge weights in the networks $G_{Q^{(n)}}$ as $n=1,5,10^{2},$ and $10^{3}$. The vertical and horizontal axes indicate edge index and edge weights, respectively. Four red circles indicate incoming edge weights of the four vertices.

Figure 3 shows the networks $G_{Q^{(n)}}$ and distributions of edge weights with increasing *n*.

**Table 1:** Rank of $\textrm{In}_{i}^{(n)}$ and the rate of change $\theta _{i}^{(n)}$ in network $G_{Q^{(n)}}$ when $n=10^{3}$.

$r_{i}^{(n)}$	Vertex	$\textrm{In}_{i}^{(n)}$	$\theta _{i}^{(n)}$
1	GOOD	$4.49\times 10^{-2}$	1.17
2	WANT	$3.81\times 10^{-2}$	1.07
3	BAD	$3.55\times 10^{-2}$	1.57
4	LOVE	$2.25\times 10^{-2}$	1.80
5	FEAR (FRIGHT)	$1.25\times 10^{-2}$	1.03
6	GRIEF	$1.21\times 10^{-2}$	1.16
7	LIKE	$1.04\times 10^{-2}$	1.00
8	HAPPY	$1.03\times 10^{-2}$	1.13
9	HATE	$9.10\times 10^{-3}$	1.01
10	DESIRE	$8.99\times 10^{-3}$	1.13
11	ANGER	$7.92\times 10^{-3}$	1.07
12	REGRET	$7.38\times 10^{-3}$	1.04
13	HOPE	$7.06\times 10^{-3}$	1.13
14	ANXIETY	$6.21\times 10^{-3}$	1.16
15	SHAME	$5.35\times 10^{-3}$	1.31
16	PITY	$4.06\times 10^{-3}$	1.11
17	ENVY	$3.64\times 10^{-3}$	1.00
18	PROUD	$3.64\times 10^{-3}$	1.89
19	SURPRISED	$1.92\times 10^{-3}$	1.12
20	SAD	$1.71\times 10^{-3}$	1.23
21	HAPPINESS	$1.39\times 10^{-3}$	1.85
22	MERRY	$7.49\times 10^{-4}$	1.75
23	JOY	$4.28\times 10^{-4}$	1.33
24	WORRY	$3.21\times 10^{-4}$	3.00
25	GLOOMY	$1.07\times 10^{-4}$	

At all networks $G_{Q^{(n)}}$ in Fig. 3, the thickness and color of the edges, namely edge weights in the network $G_{Q^{(n)}}$, change as *n* increases and converge to a constant value when $n=10^{3}$ (Fig. 3d). Moreover, the distributions of edge weights in Fig. 3c,d show that edges with the same weights exist in networks $G_{Q^{(10^{2})}}$ and $G_{Q^{(10^{3})}}$.

The network $G_{Q^{(10^{3})}}$ in Fig. 3a shows that most of the red edges with large weights are connected to the four vertices “GOOD,” “WANT,” “BAD,” and “LOVE.” These red edges were identified as the edge weights indicated by the four red circles located on the right side of the distribution of edge weights when $n=10^{3}$.

Moreover, we also found that the four red circles in Fig. 3d indentify edges with significantly large weights directed to the four vertices, or “GOOD,” “WANT,” “BAD,” and “LOVE.” This means that these four vertices are most likely to acquire edges with high weights in the emotional colexification network.

We calculated $\textrm{In}_{i}^{(10^{3})}$ and $\theta _{i}^{(10^{3})}$ of vertices in the emotional colexification network $G_{Q^{(n)}}$ when $n=10^{3}$ to confirm that the above-mentioned four vertices are hubs. Table 1 presents the rank of $\textrm{In}_{i}^{(n)}$ and $\theta _{i}^{(n)}$. In Table 1, the four concepts, “GOOD,” “WANT,” “BAD,” and “LOVE” have the largest values of $\textrm{In}_{i}^{(n)}$. Moreover, the rate of change $\theta _{i}^{(n)}$ of “LOVE” is 1.8, which is near two and the largest among the four vertices with high values of $\textrm{In}_{i}^{(n)}$. For vertex *i*, where $r_{i}^{(n)}$ is lower than four, the value of $\theta _{i}^{(n)}$ is nearly constant and close to unity. These results indicate that these four vertices have edges with high weights and can be considered hubs in the emotional colexification network. Therefore, we defined the four emotional concepts, “GOOD,” “WANT,” “BAD,” and “LOVE,” which are hubs in the emotional colexification network. Thus, we identified central emotions considered on the emotional colexification network, which are connected with a strong relevance to other concepts, namely “GOOD,” “WANT,” “BAD,” and “LOVE.”

**Figure 4:**
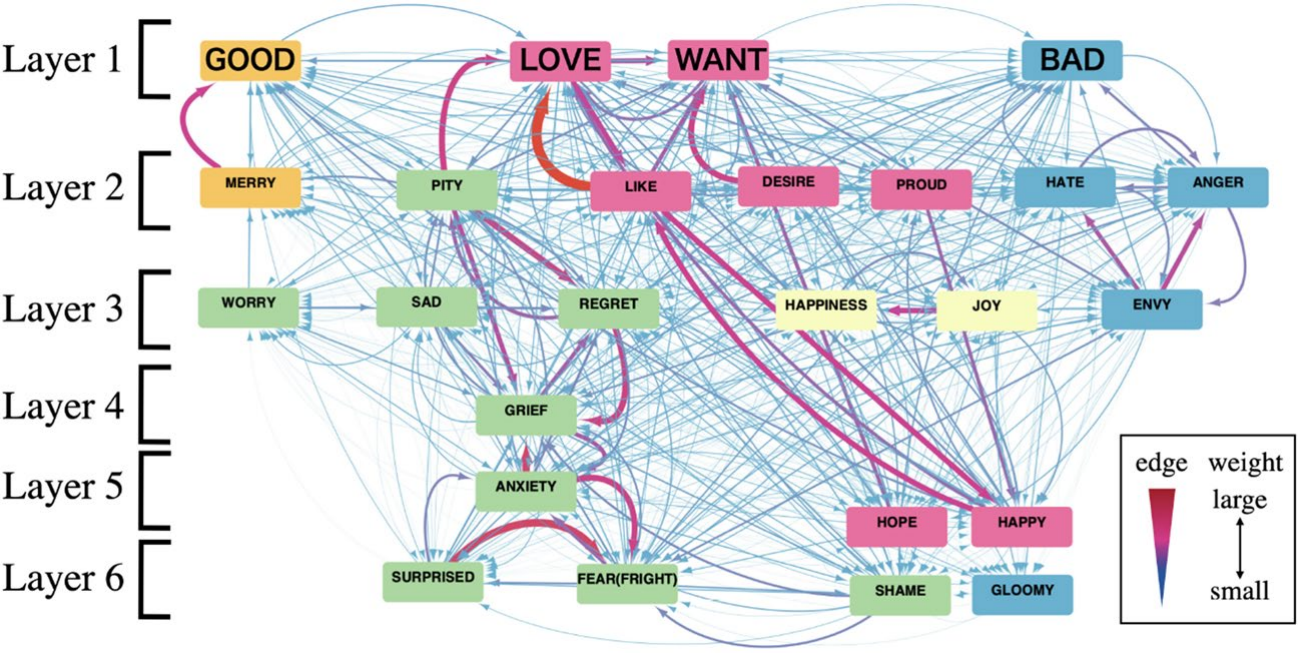
The aggregated emotional colexification network. Vertices of the same color indicate that they belong to the same community. Red edges indicate large edge weights. The thickness of the edges indicates large edge weights.

To visualize how strongly other emotional concepts have relevance to the emotional concepts corresponding to “GOOD,” “WANT,” “BAD,” and “LOVE,” we classified the network vertices into multiple layers according to the strength of their relevance with the hubs. Figure 4 shows an aggregated emotional colexification network to confirm the relationships between four hubs and other vertices.

In Fig. 4, we arranged vertices on six layers: hubs are placed on Layer 1, vertices having a strong connection with these four hubs are placed on Layer 2, and vertices having a strong connection with vertices on Layer *l* are placed on Layer $l+1~(l=1, \ldots , 5)$ (see Sec. S2 in SI text for details). The vertices belonging to the same community have the same color; five communities were identified by the community detection. The vertices in Layers 2 and 3 have high relevance for emotional concepts corresponding to the four hubs, and most of the vertices belong to the community to which the hub belongs. However, in the communities where the color of the vertices is pink or blue, concepts with lower relevance to the hub were identified such as “HOPE,” “HAPPY,” and “GLOOMY.”

Focusing on relationships between hub vertices and communities in Fig. 4, a community whose color of vertices is pink has two hubs, “LOVE” and “WANT,” whereas communities whose color of vertices is yellow and green have no hub. This indicates that in the emotional colexification network, concepts that are easily associated with many concepts and communities based on relevance are independent of each other.

**Figure 5:**
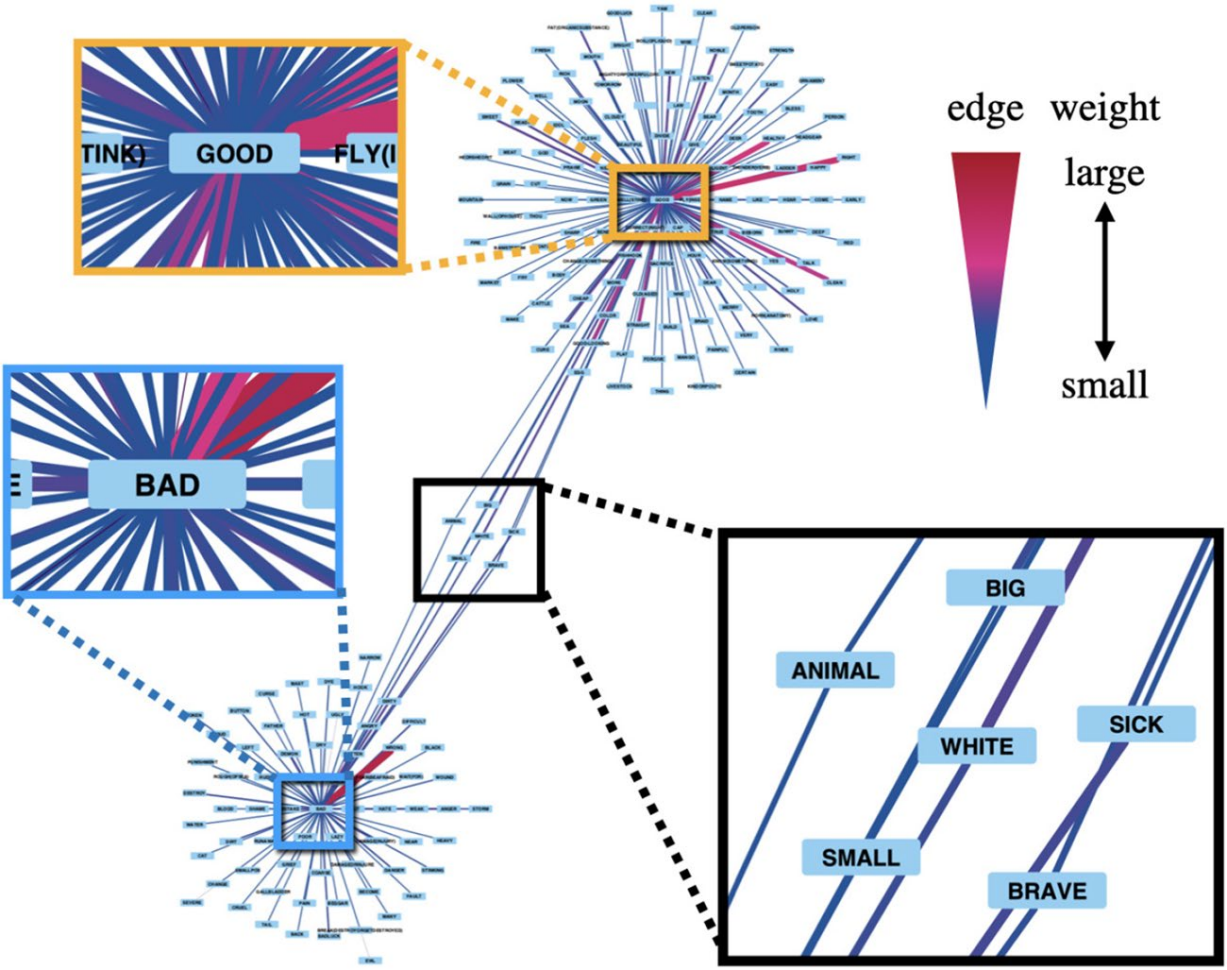
Concepts which have colexifications both “GOOD” and “BAD.” Red edges indicated large weights, and blue edges indicated small weights.

The advantage of our study is to quantify relationships not only between similar emotions but also between dissimilar emotions that are linked to each other associatively or by cooccurence. We examined the relevance between the most fundamental mutually exclusive emotional concepts: “GOOD” and “BAD.” The result in Fig. 4 shows that “GOOD” and “BAD” were mutually connected by an edge whose weight is $2.74\times 10^{-3}$ and an edge whose weight is $3.47\times 10^{-3}$ in the emotional colexification network (Fig. 4). To investigate the concepts associated with the relevance between “GOOD” and “BAD,” we have illustrated their colexifications as depicted in Fig. 5.

Figure 5 shows the results of extracting the concepts having colexifications with “GOOD” and “BAD” in the network $G_{W}$. The six concepts surrounded by the black square in Fig. 5, demonstrate colexifications with both “GOOD” and “BAD.” Among these six concepts, “BRAVE” is a concept evoked from relevance to “GOOD” and “BAD.” Furthermore, we find two elements to the English word *brave*: admirable and reckless. Hence, within the colexification network, relationships between dissimilar emotions that are linked to each other associatively or by cooccurence can indeed exist owing to the consideration of both similarity and associativity. Thus, our approach facilitates the identification of concepts that serve as bridges between emotional concepts from a new perspective.

## Discussion

We detected four hub emotions: “GOOD,” “WANT,” “BAD,” and “LOVE.” The characteristics of these central emotions are discussed below in comparison with the related concepts such as basic emotions in the fields of psychology and biology and semantic primes in the field of linguistics.

First, we compared the central emotions identified in the colexification analysis with two prominent proposals regarding the biologically basic emotions of humans. Studies of basic emotions have a long and rich history, originating from the pioneering work of Charles Darwin^10^, who argued that biologically basic emotions are characterized by typical facial expressions. Building partly on Darwin, Paul Ekman provided evidence for six basic emotions characterized by distinct facial expressions^11, 12^. Several other researchers also proposed lists of basic emotions^13–15^; however, it should be noted that the exact number and identity of the biologically basic emotions remain a subject of debate among psychologists (e.g., Ortony and Turner, 1990^16^; Ortony, 2022^17^), as is the relation of the proposed basic to nonbasic emotions (e.g., Ekman, 1992^18^; Johnson-Laird and Oatley, 1989^3^). Although our study is concerned with people’s emotional concepts rather than with emotions per se, our findings are relevant to these debates if one assumes that the prominent role that basic emotions are claimed to play by basic emotion theorists in human experience is reflected in the emotion lexicon in semantically central concepts. Based on this assumption, we compared the four central emotions detected in the colexification analysis (“GOOD,” “WANT,” “BAD,” and “LOVE”) with seven inherited emotions discussed in detail by Darwin (sadness, happiness, anger, contempt, hate, fear, and surprise) and the six basic emotions originally proposed by Ekman (happiness, sadness, anger, fear, disgust, and surprise). Evidently, there is only very limited agreement. Although “GOOD” and “BAD” can be interpreted as referring generally to, respectively, positive emotions (feeling good) and negative emotions (feeling bad) and the emotions proposed by Darwin and Ekman are either positive (happiness) or negative (all other emotions with the exception of surprise), none of the basic emotions proposed by these theorists corresponds exactly to one of our central emotions. Conversely, the states referred to by “LOVE” and “WANT” do not have counterparts in the list of basic emotions proposed by Darwin and Ekman.

Second, we compared our four central emotions to the list of semantic primes assumed in the natural semantic metalanguage (NSM) proposed by Wierzbicka and coworkers. NSM is a system of meaning representation based on a set of conceptual primitives, called semantic primes, that have been found to be encoded in numerous languages and on this basis, are assumed to be universal, by NSM theorists^19, 20^. To identify the semantic primes, the NSM researchers studied numerous languages using traditional semantic methods. Intriguingly, the set of semantic primes includes three of our four central emotion concepts: “GOOD,” “BAD,” and “WANT.” This agreement supports our conclusion that the central concepts identified by colexification analysis are shared by many languages rather than being specific to English. It also shows that the identification of the central emotion concepts is not specific to colexification analysis, because three of them were also obtained using traditional semantic analysis. The exception is “LOVE,” which we identified as a central emotion concept but which is not on the list of semantic primes. However, rather than considering this non-agreement as problematic, we believe it shows that our quantitative semantic method, or analysis by colexification networks, is able to identify possible candidates of semantic primes that have been overlooked by the traditional semantic analysis.

Although the discussion so far has been comparison of our central emotions with basic emotions and semantic primes, various other theories of emotions exist such as appraisal theories of emotions^21, 22^ and psychological constructionist theories of emotions^23^. For example, following the definition of emotion in the appraisal theory of emotions by Gendron and Barrett^21^, emotion is an act of making meaning whereas emotional concepts in the colexification network, or the central emotions, are concepts based on the meaning of words and are defined from a language perspective. On the other hand, Mun described that the psychological constructionist theory of emotions differs from the basic emotions theory and the appraisal theory of emotions, which define emotions as products of individual psychological interpretations^24^. Thus, in comparing these emotion theories, concepts of emotion in the colexification approach may help provide new insights into emotions and needs to be discussed in more depth in the future.

Furthermore, the concepts “GOOD,” “WANT,” and “BAD” that were obtained as central emotions can be also used to evaluate emotional states and in this case they do not directly represent emotions. Therefore, we additionally investigated the emotional concepts in the colexification network without these three central emotions (see Sec. S3 in SI text). The result indicates that even when the three vertices of “GOOD,” “WANT,” and “BAD” were not considered, the ranking order of the other emotional concepts remained consistent and did not fluctuate; the rate of change $\theta _{i}^{(n)}$ for “LOVE” is larger than 1.5 and is ranked as the first.

## Conclusion

Emotions have an important role in human communication. In our analysis, we specified central emotions by finding hub emotions in the emotional colexification network. We confirmed that the vertices corresponding to the following emotional concepts are central emotions: “GOOD,” “WANT,” “BAD,” and “LOVE.” Furthermore, by expressing the relationships between emotional concepts in an emotional colexification network, we clarified the relationships between emotional concepts considered not having relevance for their dissimilarity.

This study contributes to the literature on basic emotions by adding new knowledge from a novel perspective. Many studies in the field of psychology explore basic emotions only with a focus on the similarity of emotional concepts. Further, we quantified not only the similarity between concepts but also their associativity in the network. Thereby, our detected central emotions reflect the relationship between emotional concepts, not only within groups based on similarity but also across the groups.

These findings are broadly connected with the literature on natural language processing. Concepts associated with sentiments or emotions play an important role in the field of natural language processing, particularly sentiment analyses. The sentiment analysis methods enable us to identify semantically positive and negative orientations of written texts and have various applications such as identifying the sentiment orientation of product reviews^25^, predicting votes from congressional reports^26^, management research^27^, assessing damages caused by disasters^28^, and mental health applications^29, 30^. Despite these wide applications of sentiment analysis techniques, fine-grained sentiment analysis still seems to be difficult because many sentiment classifiers thus far provide only a few semantic orientations, for example, positive, negative, and neutral orientations. To obtain further fine-grained sentiment analyses, it is necessary to understand significant emotions and the inter-emotional-concept relationships discussed in this paper. In this sense, our findings can also potentially contribute to further technological advances in sentiment analysis and increase the range of its applications.

In this study, we examined the colexification of central emotions for all languages combined, i.e. without distinguishing between different languages or language families. However, as Jackson et al. show that colexification networks differ more or less between different language families, there might also be differences between languages in central emotion concepts by the colexification networks. Possible reasons for these differences are (i) that the emotion concepts include partly different meanings in different languages and (ii) that the patterns of perceived (and possibly, of actual) covariation between emotions differ between cultures (Kuppens et al., 2004^7^). To mitigate such concern, we separately examined which emotions serve as core emotions within each of the two language families: Austronesian languages and Indo-European languages. The result is that the central emotions, “GOOD,” “WANT,” “BAD,” and “LOVE” consistently appear in these colexification networks (see Sec. S4 in SI text for details).

Finally, it is important to acknowledge the limitations of colexification-based analyses of emotions. Colexification is only one facet of the semantic relationships among emotion terms, and the $\textrm{CLICS}^{3}$ database does not include all existing emotion concepts. As a consequence, the colexification networks derived from this database provide only partial information about the structure of the emotion lexicon. Future research should therefore also address the potential limitations of the colexification analysis of emotions.

### Supplementary Information

Supplementary Information.

## Data Availability

We use the newest dataset constructing $\textrm{CLICS}^{3}$ (https://clics.clld.org) as of 21st October 2021.
