# Supplementary material for: Central emotions and hubs in a colexification network
Source: Sci Rep. 2023 Dec 9;13:21823. doi: 10.1038/s41598-023-48922-8 (PMC10710517; doi:10.1038/s41598-023-48922-8)
Supplement: Supplementary file 1 — Supplementary Information. [file 41598_2023_48922_MOESM1_ESM.pdf]

Supplementary Information for Central emotions and hubs in a colexification network

Mitsuki Fukuya[1,*], **Tomoko Matsumoto**[2,+], **Yutaka Shimada**[3,+], **and Tohru Ikeguchi**[1,+]

[*]fukuya@hisenkei.net
[+]These authors contributed equally to this work
[1]Department of Information and Computer Technology, Tokyo University of Science, 6-3-1 Niijuku, Katsushika-ku, 125-8585, Tokyo, Japan
[2]Institute of Arts and Sciences, Tokyo University of Science, 1-3 Kagurazaka, Shinjuku-ku, 162-8601, Tokyo, Japan
[3]Department of Information and Computer Sciences, Saitama University, 255 Shimo-okubo Sakura-ku, Saitama-shi, 338-8570, Saitama, Japan

ABSTRACT

By focusing on colexification, we detected central emotions sharing semantic commonalities with many other emotions in terms of a semantic relationship of both similarity and associativity. In analysis, we created colexification networks from multiple languages by assigning a concept to a vertex and colexification to an edge. We identify concepts of emotions with a large weight in the colexification network and specify central emotions by finding hub emotions. Our resultant central emotions are four: "GOOD," "WANT," "BAD," and "LOVE."

Materials and Methods

S1 The definition of emotional concepts

In defining emotional concepts, we referred to the definitions of typical emotions from a previous study[1] (Ref. 5 of the main text) that created a colexifcation network using CLICS[3][2] (Ref. 8 of the main text). The labeling of the concepts corresponding to the vertices follows the concept extraction method[3] employed in CLICS[3]. In the previous study[1], 99 typical emotions were based on studies on emotions[4–13]. From these emotions, 25 emotions correspond to concepts tagged as "Emotions and values" in CLICS[3]. They were used as vertices of a colexification network. It should be noted that some concepts categorized as emotions (for example, "GOOD") in a previous study[9] do not always denote emotions directly. Instead, they might convey emotions indirectly via evaluative measures.

In Jackson et al.[1], "HAPPY" and "HAPPINESS" were consolidated into a singular emotional concept, "HAPPY." This approach was based on the assumption that both terms fundamentally represent the same emotion. In this study, the two concepts were not integrated, but were treated as different emotional concepts. Hence, the difference between them is represented in the network. Thus, 25 emotional concepts were used as the vertices of the network in this study, whereas 24 concepts were used as the vertices of the network in Jackson et al.[1]. In our emotional colexification network, "HAPPINESS" has particularly strong relevance to "JOY," whereas "HAPPY" has relevance to several emotional concepts. This shows that a bias exists in meaning even for concepts that have approximately the same meaning.

S2 How to create a flow diagram of emotions

Figure S1 shows the procedure for classifying emotional concepts into layers in order of the strength of relevance, starting from "GOOD," which is an example of a hub vertex.

First, the starting hub vertex is stored in Layer 1. Next, the vertices that have the strongest relevance to the starting hub vertex are extracted. This study performs hierarchical clustering on the edge weights towards the hub to identify the group of emotions to which the edge with the largest weight belongs, and the vertices connected by the edges belonging to this group are extracted as the vertices having the strongest relevance to the starting hub emotion and stored in Layer 2. Next, the vertices having the strongest relevance to any vertices stored in Layer 2 (strong indirect relevance to the hub) are extracted. Then, hierarchical clustering is performed on the edge weights towards any vertices in Layer 2 and extracted the vertices corresponding to emotional concepts having the strongest relevance to the emotional concepts in Layer 2. We repeated the procedure until all vertices were stored in the layers. In hierarchical clustering, this study used the Ward method[14] for linkage.

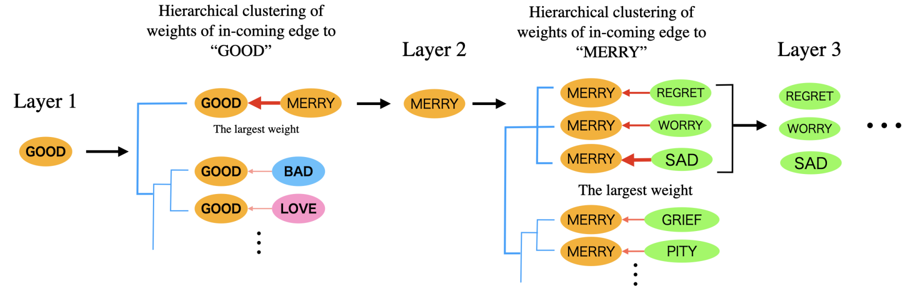

Figure S1. Example of classifying emotional concepts having strong relevance to hubs into layers.

This figure illustrates the procedure of extracting emotional concepts that have strong relevance to "GOOD," one of the four hubs, and classifying them into layers. Emotional concepts of the same color belong to the same community when community detection is performed on the emotional colexification network. First, the hub, "GOOD," is stored in Layer 1. Second, "MERRY" is stored in Layer 2 because "MERRY" has the strongest relevance with "GOOD." Third, "REGRET," "WORRY," and "SAD" are stored in Layer 3 because "SAD" has the strongest relevance with "MERRY" and because "REGRET," "WORRY," and "SAD" are classified as the same group by hierarchical clustering.

S3 Investigation of central emotions in colexification network without "GOOD," "WANT," and "BAD"

Table S1 shows the strength $\mathrm{In}_i^{(n)}$ and the rate of change $\theta_i^{(n)}$ in the colexification network in case of removing the three vertices corresponding to "GOOD," "WANT" and "BAD."

Table S1. The strength $\mathrm{In}_i^{(n)}$ and the rate of change $\theta_i^{(n)}$ in the network $G_{Q^{(n)}}$ without the three vertices corresponding to "GOOD," "WANT," and "BAD" when $n = 10^3$.

$r_i^{(n)}$	vertex	$\mathrm{In}_i^{(n)}$	$\theta_i^{(n)}$
1	LOVE	1.89×10^{-2}	1.80
2	FEAR (FRIGHT)	1.05×10^{-2}	1.03
3	GRIEF	1.01×10^{-2}	1.15
4	LIKE	8.72×10^{-3}	1.00
5	HAPPY	8.72×10^{-3}	1.14
6	HATE	7.64×10^{-3}	1.01
7	DESIRE	7.55×10^{-3}	1.13
8	ANGER	6.65×10^{-3}	1.07
9	REGRET	6.20×10^{-3}	1.04
10	HOPE	5.93×10^{-3}	1.00
11	ANXIETY	5.93×10^{-3}	1.13
12	SHAME	5.21×10^{-3}	1.16
13	PITY	4.49×10^{-3}	1.31
14	ENVY	3.41×10^{-3}	1.18
15	PROUD	3.05×10^{-3}	1.00
16	SURPRISED	3.05×10^{-3}	1.89
17	SAD	1.61×10^{-3}	1.12
18	HAPPINESS	1.43×10^{-3}	2.27
19	MERRY	6.29×10^{-4}	1.75
20	JOY	3.59×10^{-4}	1.33
21	WORRY	2.69×10^{-4}	3.02
22	GLOOMY	8.99×10^{-5}	

From Table S1, the rate of change $\theta_i^{(n)}$ of "LOVE" is 1.80 and larger than 1.5. This result indicates that the emotional concept "LOVE" is a specific central emotion in the colexification network, even if the three central concepts, "GOOD," "WANT," and "BAD," are removed from the colexification network.

S4 Differences of central emotions in colexification network between language families

To confirm whether the central emotional concepts of ("GOOD," "WANT," "BAD," and "LOVE") are also hubs in the colexification networks generated from the colexifications observed in different language families, we investigated two types of colexification networks with respect to two familiar language families: Austronesian languages and Indo-European languages. Table S2 shows the top five emotional concepts with the highest edge strength values to the vertex i (In_i) in colexification networks created from the two language families.

Table S2. The top five emotional concepts with the highest values of edge strength to the vertex i (In_i) in two language families

	Austronesian		Indo-European	
r_i	concept	$\mathrm{In}_i(\times 10^{-2})$	concept	$\mathrm{In}_i(\times 10^{-2})$
1	GOOD	3.81	BAD	4.00
2	BAD	2.57	GRIEF	2.47
3	WANT	2.21	GOOD	2.34
4	LOVE	2.04	LOVE	2.00
5	ANGER	0.97	HAPPY	1.94

From Table S2, we found that "GOOD," "WANT," "BAD," and "LOVE" may be cross-linguistic hub emotions. We will carry out the same surveys for the other language families in CLICS[3] and report differences between the language families in future studies.

References

1. J. C. Jackson, J. Watts, T. R. Henry, J. M. List, R. Forkel, P. J. Mucha, S. J. Greenhill, R. D. Gray & K. A. Lindquist, Emotion semantics show both cultural variation and universal structure. *Science*. ***366***, 1517–1522 (2019).
2. C. Rzymski, T. Tresoldi, S. J. Greenhill, M. S. Wu, N. E. Schweikhard, M. Koptjevskaja-Tamm, V. Gast, T. A. Bodt, A. Hantgan, G. A. Kaiping, S. Chang, Y. Lai, N. Morozova, H. Arjava, N. H´ubler, E. Koile, S. Pepper, M. Proos, B. V. Epps, I. Blanco, C. Hundt, S. Monakhov, K. Pianykh, S. Ramesh, R. D. Gray, R. Forkel & J. M. List, The Database of Cross-Linguistic Colexifications, reproducible analysis of cross-linguistic polysemies. *Scientific data*. ***7***, 1–12 (2020).
3. J. M. List, M. Cysouw, & R. Forkel, Conception: A Resource for the linking of concept lists in *Proceedings of the Tenth International Conference on Language Resources and Evaluation (LREC'16)* (eds. N. Calzolari, K. Choukri, T. Declerck, S. Goggi, M. Grobelnik, B. Maegaard, J. Mariani, H. Mazo, A. Moreno, J. Odijk, and S. Piperidis) 2393–2400 (European Language Resources Association, 2016).
4. P. Shaver, J. Schwartz, D. Kirson & C. O'Connor, Emotion knowledge: further exploration of a prototype approach. *Journal of Personality and Social Psychology*. ***52***, 1061–1086 (1987).
5. C. E. Salas, D. Radovic & O. H. Turnbull, Inside-out: comparing internally generated and externally generated basic emotions. *Emotion*. ***12***, 568–578 (2012).
6. P. N. Juslin & P. Laukka, Expression, perception, and induction of musical emotions: a review and a questionnaire study of everyday listening. *Journal of New Music Research*. ***33***, 217–238 (2004).
7. N. T. Feather & I. R. McKee, Differentiating emotions in relation to deserved or undeserved outcomes: a retrospective study of real-life events. *Cognition and Emotion*. ***23***, 955–977 (2009).
8. R. L. Morgan & D. Heise, Structure of emotions. *Social Psychology Quarterly*. ***51***, 19–31 (1988).
9. P. M. A. Desmet, Faces of product pleasure: 25 positive emotions in human-product interactions. *International Journal of Design*. ***6***, 1–29 (2012).
10. N. J. MacKinnon & L. J. Keating, The structure of emotions: Canada-United States comparisons. *Social Psychology Quarterly*. ***52***, 70–83 (1989).
11. J. Altarriba & L. M. Bauer, The distinctiveness of emotion concepts: a comparison between emotion, abstract, and concrete words. *The American Journal of Psychology*. ***117***, 389–410 (2004).
12. S. M. Mohammad & P. D. Turney, Crowdsourcing a word–emotion association lexicon. *Computational Intelligence*. ***29***, 436–465 (2013).
13. K. C. Berridge, Pleasures of the brain. *Brain and Cognition*. ***52***, 106–128 (2003).

14. J. H. Ward, Hierarchical grouping to optimize an objective function. *Journal of the American Statistical Association*. ***58***, 236–244 (1963).
